# 

**Published:** 1849

**Authors:** 


WHOLE SERIES—VOL. V, NO. VI.

VOL. I.] FEBRUARY AND MARCH, 1849. [No. 6.

### THE NORTH-WESTERN

### MEDICAL AND SURGICAL

### JOURNAL.

PUBLISHED EVERY OTHER MONTH.

AT TWO DOLLARS PER ANNUM IN ADVANCE.

EDITED BY

WILLIAM B. HERRICK, M.D. AND JOHN EVANS, M.D.,

PROFESSORS IN RUSH MEDICAL COLLEGE, CHICAGO.

CHICAGO AND INDIANAPOLIS:

PUBLISHED BY JOHN W. DUZAN.

1849.

